# Coexistence of weak and strong coupling in a photonic molecule through dissipative coupling to a quantum dot

**DOI:** 10.1515/nanoph-2025-0379

**Published:** 2025-11-27

**Authors:** Stefan Lichtmannecker, Santiago Echeverri-Arteaga, Michael Kaniber, Isabel C. Andrade Martelo, Joaquín Ruiz-Rivas, Thorsten Reichert, Günther Reithmaier, Per-Lennart Ardelt, Max Bichler, Eduardo Zubizarreta Casalengua, Edgar A. Gómez, Herbert Vinck-Posada, Elena del Valle, Kai Müller, Fabrice P. Laussy, Jonathan J. Finley

**Affiliations:** Walter Schottky Institut and Physik Department, 529704 Technische Universität München, Am Coulombwall 4, 85748 Garching, Germany; Programa de Física, Universidad del Quindío, 630004, Armenia, Colombia; Instituto de Física, Universidad de Antioquia, Calle 70 No. 52-21, Medellín, Colombia; Departament d’Òptica, Universitat de València, Dr. Moliner 50, 46100 Burjassot, Spain; Walter Schottky Institute, School of Computation, Information and Technology and MCQST, Technische Universität München, 85748 Garching, Germany; Departamento de Física, Universidad Nacional de Colombia, 111321, Bogotá, Colombia; Departamento de Física Teórica de la Materia Condensada, Universidad Autónoma de Madrid, 28049 Madrid, Spain; Instituto de Ciencia de Materiales de Madrid ICMM-CSIC, 28049 Madrid, Spain

**Keywords:** cavity QED, photonic molecule, quantum dot

## Abstract

We study the emission from a molecular photonic cavity formed by two proximal photonic crystal defect cavities containing a small number (<3) of In(Ga)As quantum dots. Under strong excitation, we observe photoluminescence from the bonding and antibonding modes in agreement with ab initio numerical simulations. Power dependent measurements, however, reveal an unexpected peak, emerging at an energy between the bonding and antibonding modes of the molecule. Temperature-dependent measurements indicate that this unexpected feature is photonic in origin. Time-resolved measurements show the emergent peak exhibits a lifetime *τ*_M_ = 0.75(10) ns, similar to both bonding and antibonding coupled modes. Comparisons of experimental results with quantum optical modeling suggest that this new feature arises from a coexistence of weak and strong coupling, due to the molecule emitting in an environment whose configuration permits or, on the contrary, impedes its strong coupling. This scenario is reproduced theoretically with a master equation reduced to the key ingredients of its dynamics and that roots the mechanism to a dissipative coupling between bare modes of the system. Excellent qualitative agreement is obtained between experiment and theory, showing how solid-state cavity QED can reveal intriguing new regimes of light–matter interaction.

## 1 Introduction

Cavity QED (cQED) in solid-state systems [1] quickly developed into a field of its own [2] along the Nobel prize winning precedent set by atoms in microwave cavities [3]. Unlike their atomic counterparts, solid-state systems provide great flexibility to engineer ad hoc structures in complex geometries [4]. Among the possible architectures, photonic crystal (PhC) nanostructures provide the flexibility to probe cQED phenomena in non-standard configurations [5], [6]. Due to their planar geometry, theyoffer a promising platform for future integrated quantum photonic devices [7]. High quality (Q) factors combined with the ultra-small mode volumes of PhC cavities allows cQED to be studied in the few photon limit [8], [9], [10], [11], [12], [13], [14]. Most of the cQED experiments performed to date using PhCs have been performed using a single cavity. In this work, by coupling two proximal nano-resonators to form a photonic molecule (PM) [15], [16], [17], [18], [19], [20], [21], [22], [23], [24] (for a review, see Ref. [25]), we open the way to explore new degrees of freedom with potential for entirely new functionalities. For example the energy splitting of the PM modes can be tuned via geometric parameters during fabrication or tuning using photochromic materials or nanoelectromechanical systems [26], [27], [28], [29]. This allows simultaneous enhancement of two different transitions and coupling between two quantum emitters separated by distances comparable to the optical wavelength [17], [30]. Theoretical proposals taking advantage of coupled resonators suggest new applications, such as the generation of optimized Gaussian amplitude squeezing with very small Kerr nonlinearities [31], the generation of bound photon-atom states [32] or the full optical coherent control of vacuum Rabi oscillations [33]. Photonic crystal molecules are also of great interest for solid-state implementations of photonic quantum simulators [34], [35], [36]. However, to date, only a handful of experiments have been performed using PMs, exploring nonlinear effects such as sum frequency generation [37], [38] or parametric oscillation [39], [40], despite the early demonstration of the up-conversion excitation in bulk GaAs [41], and enhanced efficiencies using planar microcavities [40], [42].

Here, we investigate the linear and nonlinear properties of an individual PM formed by two coupled PhC cavities doped with self assembled quantum dots (QDs). By performing photoluminescence (PL) and PL-excitation (PLE) spectroscopy, we provide clear evidence for the photonic coupling of the two cavities. In power dependent PL-measurements we observe bonding- (B) and antibonding- (AB) like modes of the PM at energies that are in excellent quantitative agreement with finite-difference time-domain (FDTD) simulations. Surprisingly, we observe an additional unexpected peak (W) that emerges precisely between B and AB when the system is subjected to strong excitation. Time-integrated PL measurements performed as a function of the lattice temperature and time-resolved spectroscopy reveal that this additional unexpected peak is primarily *photonic* in origin. We will show that such a peculiar phenomenology, where an anomalous peak grows in between a conventional Rabi doublet, follows from a coexistence of weak and strong-coupling, due to the molecule finding itself in an environment that either exposes or shields it from an additional decay channel which results in spoiling or preserving its coherent Rabi dynamics. A quantum-optical model that couples a QD to the PM through Markovian but correlated transitions captures this phenomenon and provides a fundamental picture of this otherwise unusual mechanism. Our result shows that the highly complex configurations one can engineer in the solid state provide interesting variations on the basic themes of light–matter interactions.

## 2 Fabrication and experiment

**Figure 1:**
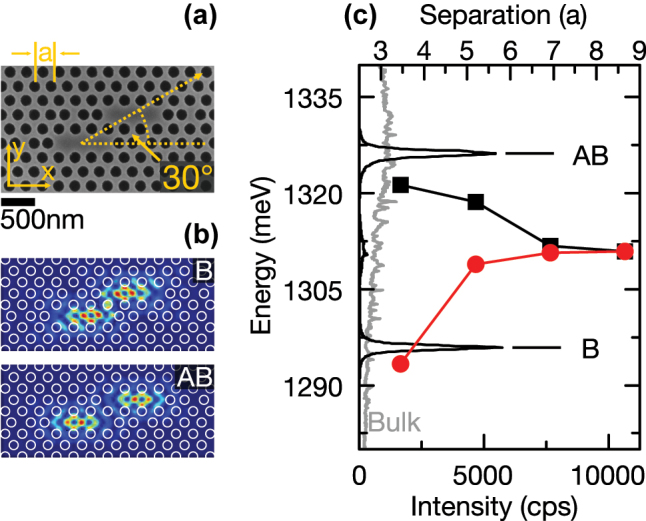
Structural characterization, simulated mode profiles and optical emission properties of the investigated PM. (a) SEM image of the PM formed by two L3 cavities. (b) |*E*|^2^ extracted from FDTD simulations for the B (upper panel) and AB (lower panel) mode. The white circles indicate the air holes forming the PhC. (c) PL spectrum of the investigated PM (black curve), and QD emission from the unpatterned region of the sample (gray curve). The solid red dots and black squares indicate the simulated energetic position of the AB and B modes as a function of cavity separation.

The sample was grown using molecular beam epitaxy on a 350 µm thick [100] orientated GaAs wafer. After depositing a 300 nm thick GaAs buffer layer, we grew a 800 nm thick sacrificial layer of Al_0.8_Ga_0.2_As, followed by a 150 nm thick nominally undoped GaAs waveguide containing a single layer of In_0.5_Ga_0.5_As QDs at its midpoint. The growth conditions used for the QD layer produce dots with an areal density *ρ*_D_ ∼ 5 µm^−2^, emitting over the energy range *E*_QD_ = 1,260 meV–1,400 meV. After growth, a hexagonal lattice of air holes with a lattice constant of *a* = 260 nm was defined in a ZEP 520-A soft mask and deeply etched using a SiCl_4_ based inductively coupled plasma to form a two-dimensional PhC. The resulting PM is formed by two L3 cavities [43] with their edges separated by a single period of the PhCl lattice as shown by the scanning electron microscopy image in Figure 1(a). In a final step, the AlGaAs layer was selectively removed with hydrofluoric acid to establish a free standing membrane.

After fabrication and characterization, the sample was cooled to a lattice temperature *T* = 13 K in a He flow-cryostat for optical study. Thereby, we used a 100× microscope objective with a numerical aperture NA = 0.8 in a confocal geometry provided by coupling the emitted signal into a single-mode fiber to spatially detect emission from a region of interest with a size of 1 μm × 1 µm. The sample was optically excited using a pulsed laser with a repetition frequency of 80 MHz. Hereby, either a non-resonant diode laser at 1,580 meV (80 ps pulse duration), or a Ti:sapphire laser with an emission energy tuned to 1,312 meV and a pulse duration of 10 ps was used. For measurements using cavity mode resonant excitation [44], [45] we used a tunable continuous-wave single-frequency laser with a bandwidth of 100 kHz. The collected emission from the sample was spectrally dispersed using an imaging monochromator with a focal length of 0.5 m and detected with a liquid nitrogen cooled CCD camera. For time-resolved measurements, a Si-avalanche photodiode was used, providing a temporal resolution of ∼350 ps without deconvolution.

For the system of two coupled L3 cavities, we expect the formation of bonding (B) and anti-bonding (AB) modes with even and odd symmetry, respectively [18]. FDTD simulations [46] for cavities having different separations and relative orientations revealed that the 30° configuration between the L3-cavity axis and a line connecting the cavity centers provides the strongest coupling for a given nominal separation between cavity centers [18]. Simulations using geometrical parameters extracted from the scanning electron microscopy image shown in Figure 1(a) yield the electric field distribution of the B and AB modes and their relative energies presented in Figure 1(b). The energy splitting between these two modes is plotted in Figure 1(c) versus the cavity–cavity separation. For a cavity separation of one row of air holes, we expect an energy splitting of $\Delta E_{sp}^{th}=$ 28 meV. For comparison, we plot in Figure 1(c) the PL emission recorded from the investigated PM (black curve) using strong excitation and the QD emission from an unpatterned region of the sample as a reference (gray curve). We clearly observe emission from the B and AB modes with an energy splitting of $E_{sp}^{\exp}=$ 30.0(1) meV, in fair quantitative agreement with our simulations [18], [19], [20]. The Q-factors of the B and AB modes were measured to be ∼1,700 and ∼1,400 , respectively. Both cavities are always excited simultaneously, as the cavity–cavity separation is smaller than the excitation laser spot of 1.1(1) µm.

## 3 Results and discussions

**Figure 2:**
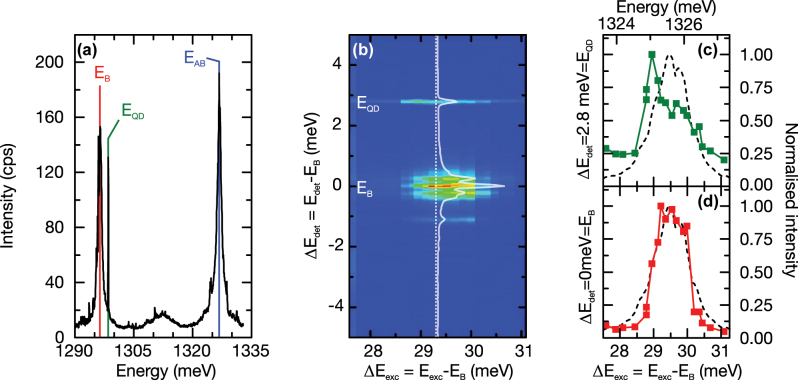
Optical characterization of the PM. (a) PL emission spectrum of the PM under pulsed above-bandgap excitation. On the high energy side of the B mode, the emission line of a QD, depicted in green, is observed with a detuning of Δ*E*_QD_ = 2.8 meV. The corresponding energies are labeled with B, QD, and AB. (b) Emission intensity as a function of excitation laser energy, whilst tuning the laser across the antibonding resonance. The white line shows an emission spectrum for the excitation energy marked by the white dotted line. (c) Emitted intensity at the QD energy at Δ*E*_det_ = 2.8 meV as a function of laser detuning. The dashed line shows PL emission of the AB mode subjected to non-resonant excitation for comparison. (d) Emitted intensity of the bonding mode at Δ*E*_det_ = 0 meV. The dashed line shows PL emission of the AB subjected to non-resonant excitation mode for comparison.

In Figure 2(a) we present a typical μ-PL spectrum recorded from the PM under pulsed non-resonant excitation. Again, we observe the emission of the B and AB modes as well as a sharp emission line attributed to a single QD at 1,298.5 meV, depicted in green, with a detuning of Δ*E*_QD_ = *E*_QD_ − *E*_B_ = 2.8 meV relative to the energy of the B mode (*E*_B_). The respective relative energies of the B mode *E*_B_ (red), the QD *E*_QD_ (green) and the AB mode *E*_AB_ (blue) are labeled on the figure. To demonstrate that spatially delocalized molecular-like modes are formed in the PM, we excited the system at resonance at the AB mode energy. This allows us to directly pump the cavity mode and excite QDs that are located at positions close to the electric field antinodes within either of the two cavities [44], [45]. To do this, we tuned a single frequency laser across the emission energy of the AB mode from 1,327.2 meV to 1,324.0 meV in steps of 200 µeV. Simultaneously, we detected emission spectra in the spectral vicinity of the B mode. Figure 2(b) shows the color-coded emission intensity as a function of the detection energy relative to the B mode, Δ*E*_det_ = *E*_det_ − *E*_B_, and the excitation laser energy Δ*E*_exc_ = *E*_exc_ − *E*_B_, for a pump power density of 94.5 W cm^−2^. We observe two clear maxima at Δ*E*_det_ = 2.8 meV and Δ*E*_det_ = 0 meV when resonantly exciting via the AB mode (Δ*E*_exc_ = 29.3 meV) attributed to the QD and the B mode, respectively. The white line shows an emission spectrum for the resonance condition Δ*E*_exc_ = 29.3 meV indicating that the QD and the bonding mode are simultaneously excited via the higher-energy AB mode. In Figure 2(c) and (d), we compare horizontal cross-sections through the QD and B mode emission at Δ*E*_det_ = 2.8 meV and Δ*E*_det_ = 0 meV, respectively. For both detection energies, we simultaneously observe a clear maximum when resonantly exciting via AB. The dashed black lines show the PL spectrum of the AB mode for comparison. The observation of a shared absorption resonance for both the QD and B mode confirms that the two cavities are indeed coupled and that the QD is spatially coupled to one of the two cavities forming the PM.

**Figure 3:**
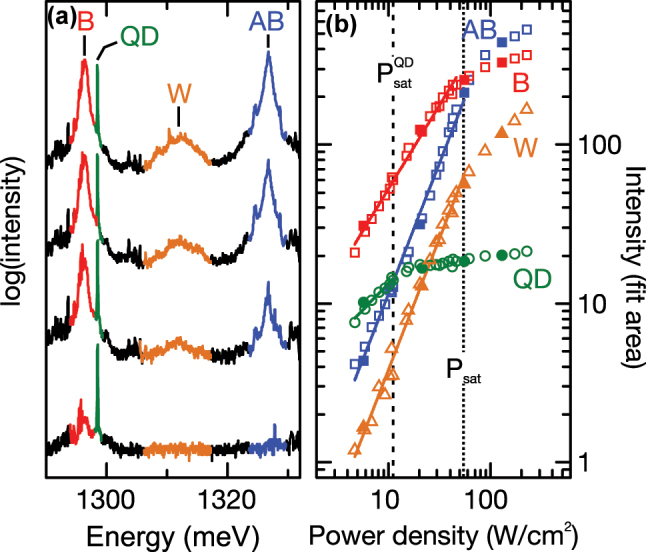
Photoluminescence and power-dependence response of the PM under non-resonant excitation. (a) PL intensity of the investigated PM plotted on a logarithmic scale, under pulsed non-resonant excitation. (b) Extracted integrated intensities of B, AB, QD and W as a function of the pump power density, shown in double logarithmic representation. The excitation power densities for the plotted spectra in (a) are highlighted with filled symbols. The black dashed and dotted lines show the saturation power density $P_{sat}^{QD}$ of the QD and the common saturation power *P*_sat_ of the B, AB and W peaks, respectively. The solid lines represent power-law fits to the data points.

After confirming the coupled character of the two cavities forming the PM, we present detailed investigations of the linear and nonlinear optical properties of the PM coupled to the QD. Figure 3(a) shows typical emission spectra from the PM subject to non-resonant pulsed excitation at 1,580 meV as the excitation power density is increased from ∼2 W cm^−2^ to >200 W cm^−2^. As discussed already in the previous paragraph, we observe pronounced emission from B, AB and the QD. More strikingly, an additional emission feature, labeled W in Figure 3(a), emerges for elevated excitation power densities. The unexpected W emission is energetically centered precisely between B and AB at *E*_M_ = 1,311 meV ≈ (*E*_AB_ + *E*_B_)/2. In Figure 3(b) we present the integrated peak intensities of B, AB, QD and W as a function of the excitation power density, plotted on a double logarithmic representation. The filled symbols label the excitation power densities selected for the spectra plotted in Figure 3(a). The QD transition increases sublinearly with excitation power density, followed by saturation of the emission for excitation power densities above $P_{sat}^{QD}=$ 13(2) W cm^−2^, as indicated by the green line in Figure 3(b). The B mode increases linearly with an exponent of 1.07 ± 0.02, due to non-resonant feeding via QD ground states [47], [48], [49], [50]. In contrast, we observe for the AB mode a clear superlinear increase in intensity of 1.65 ± 0.03, most likely arising from its proximity to excited QD states. This attribution is supported by time-resolved measurements discussed in detail in Figure 5(a). Both A and AB modes saturate at comparable power densities of *P*_sat_ = 54(4) W cm^−2^ highlighted with the dotted line in Figure 3(b). For the unexpected W peak, we observe a super-linear exponent of 1.68 ± 0.03, despite being at lower energy than expected for the QD excited states. Moreover, the W peak exhibits a similar saturation power density *P*_sat_ = 54(4) W cm^−2^ as the B and AB modes. This excitation power is 4.3× higher than the saturation power $P_{sat}^{QD}$ observed for the QD.

**Figure 4:**
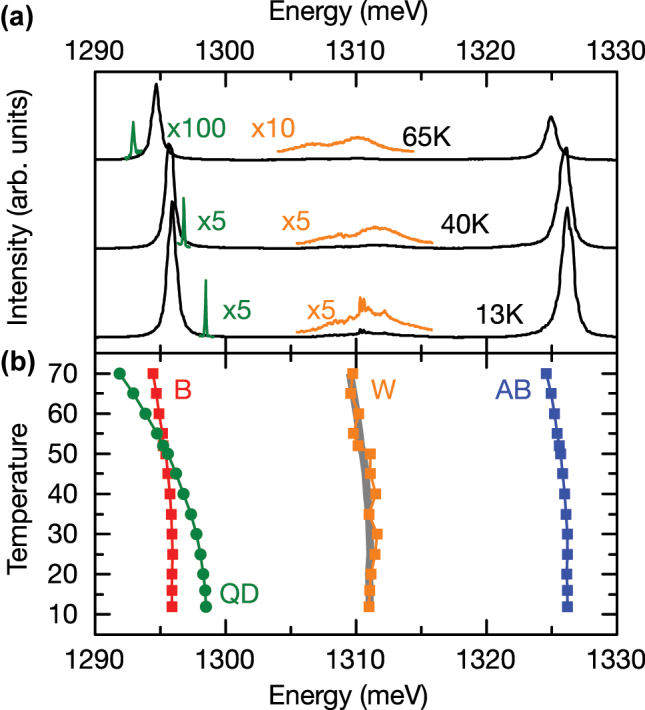
Temperature-dependent photoluminescence. (a) PL emission of the PM for selected temperatures with an excitation power density of 99 W cm^−2^ (black curves), magnified W peak (orange curves) and the QD emission under an excitation power of 3.2 W cm^−2^ (green curves). (b) Peak energies of the B mode (red), the QD (green), the W (orange) and the AB mode (blue) as a function of crystal temperature. Gray shaded region marks the calculated center between B and AB.

In order to shed light on the origin of the W peak, we present, in the following, temperature-dependent PL measurements under non-resonant CW excitation which enable us to clearly distinguish between the QD-excitonic or photonic character of the individual emission features. For the cavity modes, we expect a weak and approximately linear shift with increasing temperature, due to the change in the refractive index with increasing temperature [51]. In contrast, the QD is expected to exhibit a significantly stronger shift determined by a Varshni type relation *E*_gap_(*T*) = *E*_gap_(*T* = 0) − (*αT* ^2^)/(*T* + *β*), where *α* and *β* are dependent on the material. For GaAs, *α* = 8.871 × 10^−4^, *β* = 572 [52] and *E*_gap_(*T* = 0) = 1,521.6 meV [53], [54] and for InAs, *α* = 3.158 × 10^−4^, *β* = 93 and *E*_gap_(*T* = 0) = 426 meV [52], [55]. In Figure 4(a), we present PL spectra recorded from the cavity modes (black curves) and a magnified region around W (orange curves), as well as the QD emission (green curves) for three selected crystal temperatures, 13 K, 40 K and 65 K at two excitation power densities; 99 W cm^−2^ (black and orange curves) and 3.2 W cm^−2^ (green curves), respectively. For both cavity modes and the QD, we observe clear shifts to lower energy with increasing lattice temperature. However, the QD exhibits a higher shift rate. In Figure 4(b), we present the extracted peak positions of the different emission lines whilst tuning the sample temperature from 13 K to 70 K in steps of 5 K. For the QD, we obtain a clear nonlinear shift of the emission with temperature, yielding an average shift rate of 113.6 μeV K^−1^. As expected, the average shift rates for the cavities modes A and AB are 25.4 μeV K^−1^ and 27.6 μeV K^−1^, respectively, and thus a factor × 4.3 smaller as compared to the QD. The pronounced difference in shift rates for QD and B leads to a clear resonance for a temperature of *T* = 52 K. We observe that the W peak (orange) shows an average shift of 21.1 μeV K^−1^, similar to B and AB and stays centered between both modes over the whole temperature range, as supported by the calculated center energy *E*_M_ shown in gray in Figure 4(b). This demonstrates that the observed peak W is predominantly photonic-like and most likely does not arise from excitonic QD states.

**Figure 5:**
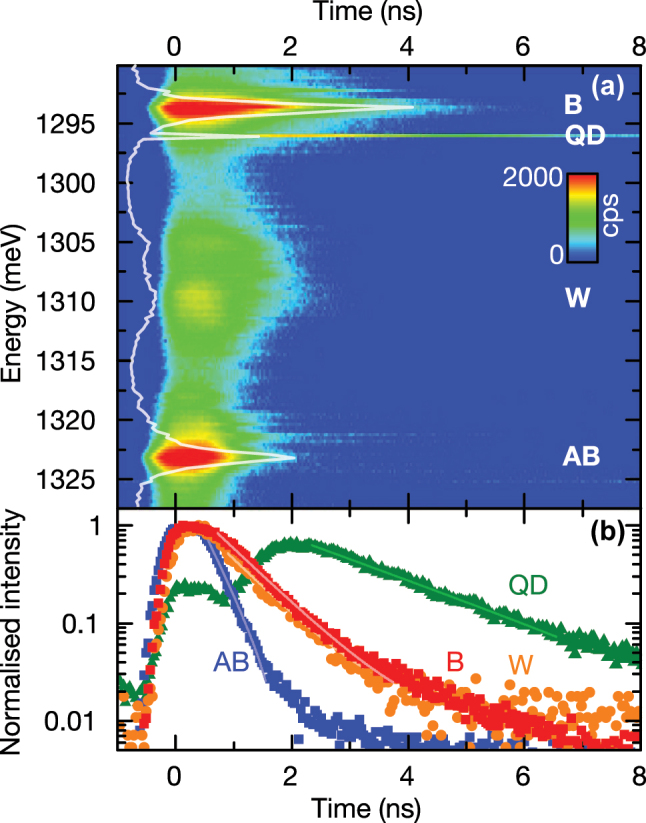
Energy and time-resolved μ-PL measurements of the PM. (a) The color scale indicates the intensity of the recorded PL signal increasing from blue to red. The white line represents the time-integrated intensity over all collected times. (b) Selected decay transients for AB, B, QD and W under non-resonant excitation.

Before suggesting compelling evidence for the nature of the unexpected W peak by comparing our results with a theoretical model, we continue to explore the decay dynamics of the coupled QD-PM using time-resolved spectroscopy. Measurements were performed as a function of emission energy (1,290 meV < *E* < 1,327 meV) subject to non-resonant excitation at 1,579 meV and a repetition frequency of 80 MHz. We used a spectrometer as tunable Δ = 270 µeV bandpass filter and recorded time transients using a time-correlated single-photon counting module [48]. Figure 5(a) shows the complete detection energy- and time-resolved PL map. The white curve represents the time-integrated signal over all recorded times and resembles the typical PL spectra recorded with a CCD camera. We clearly observe the B and AB modes, as well as the QD that shows a ∼3 times slower decay and, thus, is still visible in the time transient when the signal of the cavity modes have completely decayed. In Figure 5(b), we present selected decay transients of the B (red) and AB (blue) modes, the QD (green), as well as the W (orange) peak, as labeled in Figure 5(a). Both the B and AB modes exhibit fast decays, from which we extract lifetimes of *τ*_B_ = 0.76(10) ns and *τ*_AB_ = 0.35(10) ns, respectively. The shorter lifetime for the AB cavity mode is most likely caused by the spectral overlap with excited QD states [48]. For the QD emission we observe a step-like increase in intensity as shown in Figure 5(b), accompanied by a delayed onset of the luminescence decay. Moreover, a clear anti-correlation between the AB and the QD signal is observed; the AB mode has fully decayed prior to the QD decay. Both observations strongly suggest that the AB mode is predominantly fed from energetically higher excited multi-exciton states [56]. The exciton lifetime from the delayed QD decay yields *τ*_QD_ = 2.0(1) ns. The W peak, represented by the orange symbols in Figure 5(b), shows a lifetime similar to the B and AB modes with *τ*_M_ = 0.75(10) ns, supporting again our conclusion of the photonic-like origin of the W emission.

**Figure 6:**
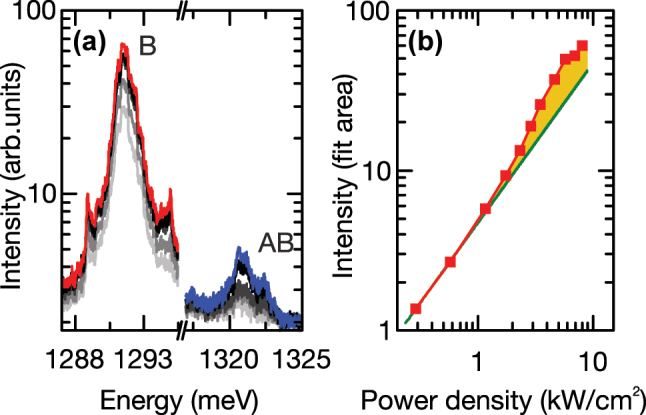
Resonant excitation of the PM at the W peak. (a) μ-PL spectra detected from the PM under excitation with 10 ps pulses resonant to the W peak at 1,311 meV for different optical pump powers. (b) PL intensity of AB as a function of excitation power density in double logarithmic representation depicted in red. The green line shows a linear power-law fitted to the first data points. The yellow area highlights the nonlinear fraction of the emission.

Finally, we performed pulsed resonant excitation of the W mode and power densities increasing from 290 W cm^−2^ up to 8 kW cm^−2^. The result, shown in Figure 6(a), clearly shows emission from both B and AB. Although the excitation laser is tuned to lower energy than the AB mode, we detect significant emission from the higher energy AB mode (blue). The B mode can be directly excited via linear absorption of the excited states of the QD, since the laser is tuned to higher energy than the emission. In Figure 6(b), we plot the PL intensity of AB as a function of the excitation power density. For low excitation power densities (*P* < 1 kW cm^−2^), we observe a linear dependence of the emission from AB with an exponent of 1.04 ± 0.04. However, for higher excitation power densities (*P* > 2.5 kW cm^−2^), the emission becomes super-linear, with the yellow shaded region highlighting the difference between the linear and super-linear behavior. This indicates that the W mode is directly coupled to the B and AB modes, but there is *a priori* no mechanism to account for this coupling. In the following, we will discuss how this can arise from a coexistence of weak and strong coupling of the molecule.

## 4 Identification of the anomalous peak

The observation of a triplet peak structure in solid-state strong-coupling experiments [57], [58], [59], [60] has been a recurrent conundrum for theorists [61], [62], [63]. Like in some other cases where a spectral triplet was observed when only a Rabi doublet was expected, our explanation relies on a mixture of weak and strong coupling. But instead of a mere incoherent superposition of the two regimes, that would be observed independently in separate time windows, our system involves an inextricable coexistence where both the weak and strong couplings occur simultaneously or at least during the smallest timescale of the system dynamics. In our case, the puzzle involves a strong, efficient and strongly asymmetric coupling mechanism already characterized in such solid-state platforms [64]. The molecule finds itself in either one these two scenarios: with the QD that excited it in the first place, likely through a phonon-assisted process, now in its ground state, or, on the contrary, still in its excited state. Both situations are possible because there is a probabilistic aspect to both the excitation and emission of the various components, so that the QD can be re-excited before the molecule gets de-excited. However, this incoherent transfer of excitation can be correlated, and this is a crucial element for coexistence of the two types of coupling. If the QD is de-excited, the molecule finds in the empty QD an efficient decay channel that brings it in weak coupling. On the other hand, if the QD is still excited, the probability for the molecule to decay through this channel gets suppressed and it therefore retains its strong coupling. We provide a simple theoretical model that produces this rich and unexpected phenomenology according to the mechanism we have just described. Unlike the model based on the multi-excitonic structure of the QD [61], our mechanism holds with a simple two-level system. The Hamiltonian itself is the simplest possible one to capture the key dynamics of our system: two cavities *a* and *b* coupled with a strength *g* much larger than the coupling *g*_*σ*_ of a QD *σ* coupled to one cavity only. The Hamiltonian describing this situation is so far standard and reads

(1) $$\begin{array}{ll} H & =\omega_{a}a^{†}a+\omega_{b}b^{†}b+\omega_{\sigma }\sigma^{†}\sigma +g\left(a^{†}b+b^{†}a\right)\\ & \;+g_{\sigma }\left(a^{†}\sigma +\sigma^{†}a\right). \end{array}$$

**Figure 7:**
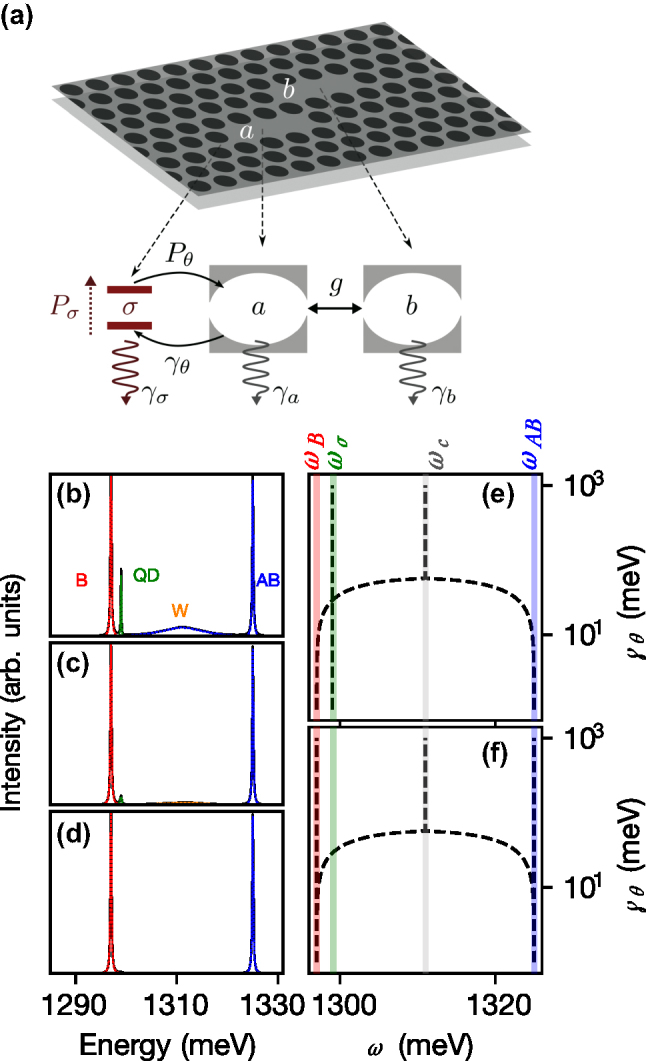
Theoretical modeling with Liouvillian eigenvalues in the linear and nonlinear regime. (a) Sketch of the model describing the system: a QD *σ* coupled to cavity *a*, itself part of a photonic molecule with cavity *b*. The straight solid double arrows correspond to the Hamiltonian coherent couplings *g* and *g*_*σ*_, while the curved solid lines are the Liouvillian dissipative coupling that we attribute to phonon-mediated transitions. The dotted line is the incoherent pumping and wavy lines are the decay terms. (b–d) PM emission spectrum (incoherent sum of both cavity spectra) as a function of pumping (solid black lines) and its decomposition in terms of the various modes involved (colored lines). (e–f) Imaginary parts of the Liouvillian eigenvalues for (e) the first manifold of excitation (linear regime) and (f) the two-excitation manifold (nonlinear regime) computed numerically but matching excellently the analytical expressions Eqs. (3) and (4), respectively. Panel (f) shows the coexistence of weak and strong coupling through the concomitance of their respective eigenvalues. The parameters used are *g* = 14 meV, *g*_JC_ = 0.14 meV, *γ*_*a*_ = *γ*_*b*_ = 0.23 meV, *γ*_*σ*_ = 0.001 meV, *P*_*θ*_ = 0.02 meV, *γ*_*θ*_ = 100 meV and *ω*_a_ = *ω*_b_ = 1311 meV, *ω*_a_ = *ω*_b_ = 1299 meV, for panel (b) *P*_*σ*_ = 0.1 meV, for panel (c) *P*_*σ*_ = 0.3 meV, for panel (d) *P*_*σ*_ = 0.9 meV for panels (e)–(f) *P*_*σ*_ = 0.

The dynamics is described with a master equation $\partial_{t}\rho =L\rho$ for the total density matrix *ρ*, where the Liouvillian L takes the form:

(2) $$\begin{array}{ll} L\rho & \equiv i\left[\rho ,H\right]\\ & \;+\left\{\gamma_{a}L_{a}+\gamma_{b}L_{b}+\gamma_{\sigma }L_{\sigma }+P_{\sigma }L_{\sigma^{†}}\right.\\ & \;\left.+P_{\theta }L_{\sigma a^{†}}+\gamma_{\theta }L_{\sigma^{†}a}\right\}\rho , \end{array}$$

where $L_{c}\rho$ is the superoperator that is defined, for a generic operator *c*, as *c* ^†^ *cρ* + *ρc* ^†^ *c* − 2*cρc* ^†^. Equation (2) describes, respectively, the cavities *a* and *b* lifetime, the QD lifetime and rate of excitation and, crucially, an incoherent coupling mechanism between the QD and cavity *a* leading to the excitation of the cavity by the QD (at rate *P*_*θ*_) or on the opposite to its de-excitation by transferring back the excitation to the QD (at rate *γ*_*θ*_). Importantly, this phonon-cavity coupling is correlated as arising from a simultaneous transfer of the excitation from the QD to the cavity, or vice-versa, as mediated by a phonon. These terms arise for instance from the phonon-mediated coupling studied experimentally and modelled theoretically by Majumdar *et al*. [65] to account for this type of cavity feeding in microcavity QED. This dissipative but correlated mechanism transfers not only excitations but also maintains or even establishes new coherent channels between the modes, which can lead to unexpected resonances. This happens even when coupling two harmonic oscillators, where it was shown to enable spontaneous coherence buildup [66]. It was also shown to provide an alternative explanation for the spectral triplet in the usual case of light–matter (Jaynes–Cummings) coupling [67], where the interpretation is then in terms of a dynamical phase transition of all the rungs from the Jaynes–Cummings dissipative ladder, as its nonlinearities eventually coalesce. There too, phonon-assisted transitions were identified as the most likely to provide this type of dissipative coupling mechanism [68]. A sketch of our model is shown in Figure 7(a). Note that the incoherent version of the QD-‘cavity *a*’ coupling allows different rates of excitation transfers, unlike the Hamiltonian case where the flow back and forth has the same rate *g*_*σ*_. This is actually one of the important features of the model as the W peak is produced in conditions where *γ*_*θ*_ ≫ *P*_*θ*_. In fact, this condition is more important for producing a state-dependent configuration of the molecule emission than the saturable two-level character of the QD. The Diagonalisation of the Liouvillian [69], [70] can be obtained in closed-form for the lowest order of the excitation manifolds in the limit *g*_*σ*_ → 0 as:

(3a) $$\begin{array}{ll} \lambda_{1,2} & \approx i\omega_{c}\pm \frac{i}{4}\sqrt{16g^{2}-{\left(\gamma_{a}-\gamma_{b}+\gamma_{\theta }\right)}^{2}}\\ & \;-P_{\sigma }-\frac{\gamma_{a}+\gamma_{b}+\gamma_{\theta }}{4}, \end{array}$$

(3b) $$\lambda_{3}\approx i\omega_{\sigma }-\frac{P_{\theta }+P_{\sigma }+\gamma_{\sigma }}{2}.$$

The case *g*_*σ*_ ≠ 0 is non-analytical but we have checked numerically that in the parameter range of interest, the structure is well described by the approximations (3), which furthermore support the observed phenomenology. The imaginary parts provide the three resonances at which the system emits, which are the A, AB and QD lines, respectively, as indeed observed in the corresponding linear regime of low-excitation. In this case, increasing dissipation and dephasing leads to the loss of strong coupling, according to the standard scenario of light–matter coupling.

The next manifold of excitation, which plays a role in the nonlinear regime when multiphoton dynamics sets in, brings an interesting variation for the structure of the eigenvalues, although this time we also need to set pumping terms *P*_*σ*_ → 0 and *P*_*θ*_ → 0 as well as *γ*_*σ*_ → 0 to retain analytical expressions. There again, we checked numerically that the results remain well described by the approximations in these limits, that capture the mechanism at play:

(4a) $$\begin{array}{ll} \lambda_{4,5} & \approx i\omega_{c}\pm \frac{i}{4}\sqrt{16g^{2}-{\left(\gamma_{a}-\gamma_{b}+\gamma_{\theta }\right)}^{2}}\\ & \;-\frac{3\left(\gamma_{a}+\gamma_{b}\right)}{2}-\frac{3\gamma_{\theta }}{4}, \end{array}$$

(4b) $$\begin{array}{ll} \lambda_{6,7} & \approx i\omega_{c}\pm \frac{i}{4}\sqrt{16g^{2}-{\left(\gamma_{a}-\gamma_{b}\right)}^{2}}\\ & \;-\frac{3\left(\gamma_{a}+\gamma_{b}\right)}{2}. \end{array}$$

The first set of eigenvalues, Eq. (4a), is the standard Rabi splitting between the A and AB modes with the same fragility to dissipation, in effect, the imaginary parts of *λ*_4,5_ being identical to those of *λ*_1,2_ and differing only in their real parts that describe broadening of the lines. The second expression, however, Eq. (4b) has no contribution from the dissipative coupling *γ*_*θ*_. This is due to the correlated character of this incoherent coupling between the QD and the PM. If the QD is excited then the term *σ* ^†^ *a* vanishes altogether and the coupling, although still sensible to the other channels of dissipation (*γ*_*a*_ and *γ*_*b*_), is now shielded from the phonon one. In cases where *γ*_*θ*_ ≫ *γ*_*a*_, *γ*_*b*_ and the QD is in its ground state, the phonon-assisted mechanism plays a role and its added dissipation overcomes the Rabi oscillations of the PM; this results in the coexistence of the two channels. This provides a quadruplet structure for the emission spectrum. However, since the broadening corresponding to the weaker *λ*_4,5_-splitting is larger than that corresponding to *λ*_6,5_, this makes difficult to resolve spectrally four peaks. Instead, one obtains features of a weakly-coupled system in the form of a broad single central peak. This is the structure we observe in the experiment as the W peak, although with hindsight, one could also recognize signs of a quadruplet for instance in Figures 4(a) and 5(a). There, a doublet is apparent, although it is of imbalanced height, just as, however, the outer doublet (which could be due to a slight detuning or other variations from an ideal light–matter coupling scenario).

The necessity in the model of the correlated character for the excitation transfer between the QD and the PM as well as the existence of two Rabi splittings depending on the state of the QD, suggest an analogy with phonon-sidebands, that are produced as the result of an optical transition affecting its surrounding matrix. Although phonons are also likely responsible in our case for making this scenario possible, the surrounding matrix itself is actually the QD and we have therefore a 0D counterpart of this phenomenon. It is enough to focus on the dynamics of the first two manifolds of excitation for such a system which is essentially that of two coupled oscillators. Higher manifolds maintain essentially this structure with small multiplicities of the resonances gathering around the skeleton of Eqs. (3) and (4). We also find that odd-numbered photon transitions, i.e., from 2*k* + 1 excitations to 2*k* for *k* ≥ 0, including the linear regime, feature the QD transition, while those with even-numbered photons, from 2*k* photons to 2*k* − 1 for *k* ≥ 1, do not feature it. Another refinement of the mechanism is that the small deviations from manifold to manifold coalesce at the critical dissipative coupling rate $\gamma_{\theta }^{c}=4g+\gamma_{b}-\gamma_{a}$ , as was previously described for the Jaynes–Cummings ladder [67], [71]. There, nonlinearities were however much larger and thus with strong manifold-to-manifold deviations, making the so-called dynamical phase transition compelling, while it remains here covert due to the dominating harmonicity of the PM, that captures the mechanism at the lowest multiphoton (nonlinear) manifold.

**Figure 8:**
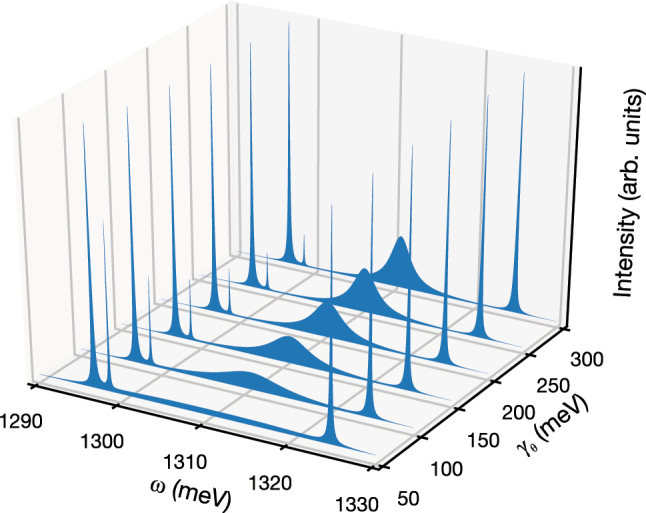
Calculated PM emission spectrum (incoherent sump of both cavity spectra) of the system as a function of *γ*_*θ*_, for the parameters *g* = 14 meV, *g*_JC_ = 0.14 meV, *γ*_*a*_ = *γ*_*b*_ = 0.23 meV, *γ*_*σ*_ = 0.001 meV, *P*_*θ*_ = 0.08 meV, *P*_*σ*_ = 0.08 meV, *E*_*g*_(0) = 1,299.0 meV and *ω*_*c*_(*T* = 0) = 1,311 meV.

**Figure 9:**
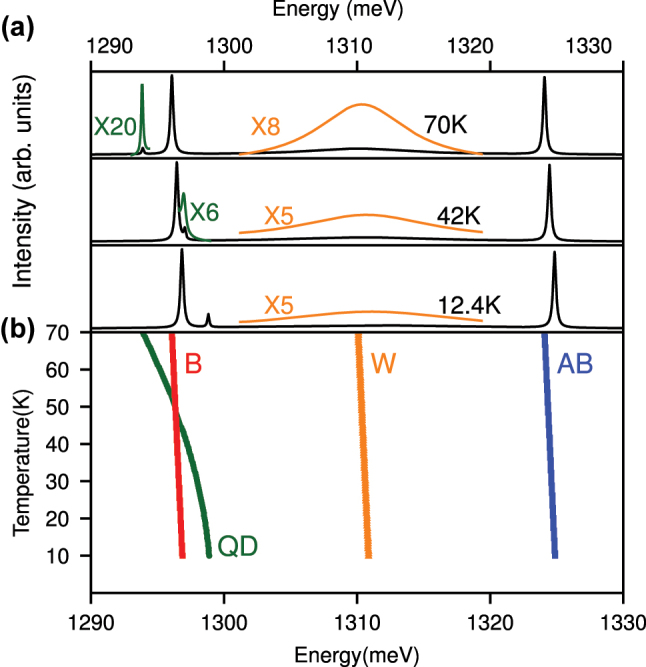
Emission spectrum of the PM (incoherent sum of both cavity spectra) at different temperatures (compare experimental data in Figure 4). Panel (a) *T* = 70 K, *T* = 42 K and *T* = 12.4 K. Additionally, panel (b) shows the center of the peaks as a function of the temperature for the parameters *g* = 14 meV, *g*_*σ*_ = 0.14 meV, *γ*_*a*_ = *γ*_*b*_ = 0.23 meV, *γ*_*σ*_ = 0.001 meV, *P*_*θ*_ = 0.08 meV, $\gamma_{\theta }\left(T\right)=60+\frac{40}{1+12e^{-0.15\left(T-0.15\right)}}$ , *E*_*g*_(0) = 1,299.0 meV, *ω*_*c*_(*T* = 0) = 1,311 meV and *P*_*σ*_ = 0.08 meV.

**Figure 10:**
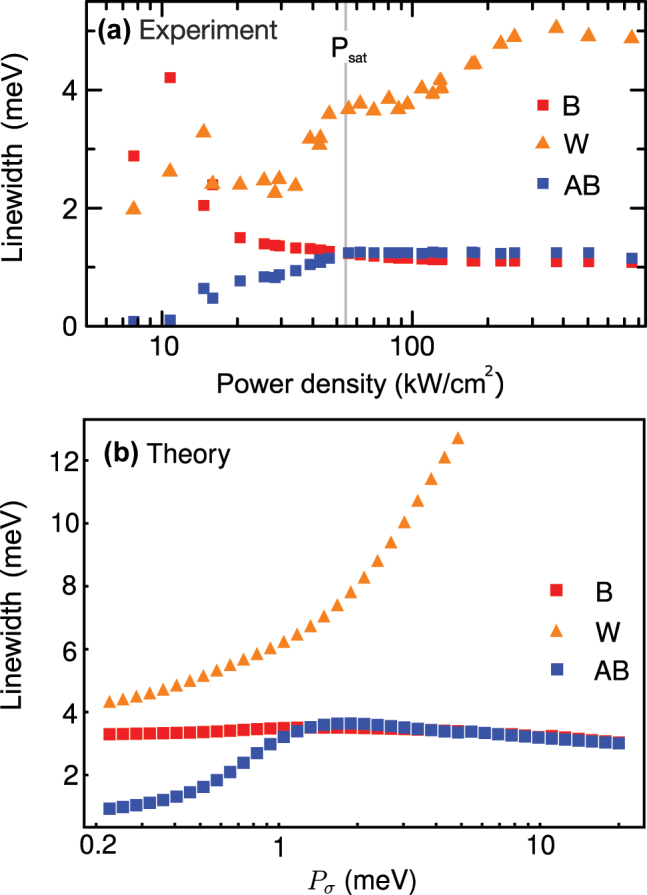
Linewidths as a function of the power density of the three emission peaks. The labels B indicates bonding as red squares, AB the anti-bonding as blue squares and W central peak as orange triangles. The quantum dot is in resonance with the bonding mode. Experimental measurements are shown in panel (a) for pulsed excitation (compare Figure 3), where the gray line highlights the saturation power *P*_sat_ of the B and AB cavity modes. The numerical calculations based on our theoretical model are shown in panel (b) where the parameters used are *g* = 14 meV, *g*_JC_ = 0.14 meV, *γ*_*a*_ = *γ*_*b*_ = 2.3 meV, *γ*_*σ*_ = 0.6 meV, *P*_*θ*_ = 0.001 meV, *γ*_*θ*_ = 557 meV and *T* = 70 K.

**Figure 11:**
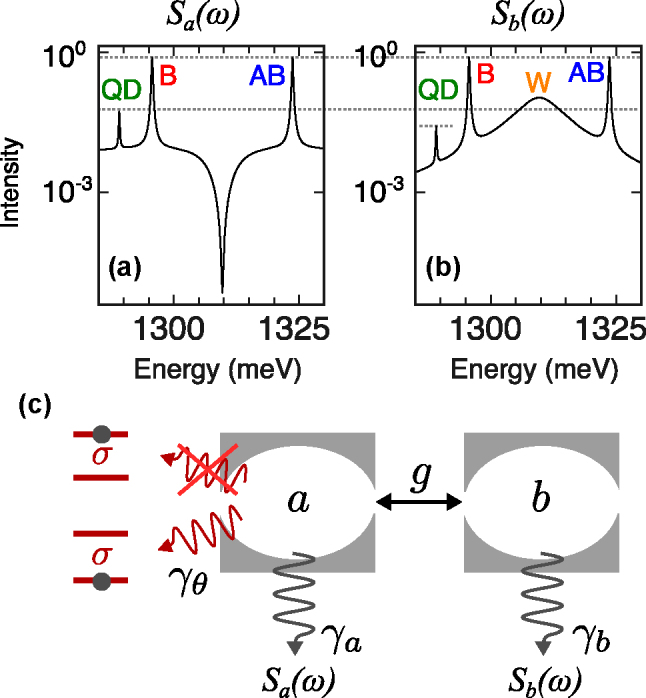
Further qualitative manifestations of the mechanism (theory only). The strong correlations from the dissipative coupling result in a depletion of bare photon emission in the cavity (a) that couples to the QD, while the loss of strong-coupling is observed as the W peak in the other cavity (b). Consequently, the QD emission is also stronger as perceived through the cavity *a* where the transfer is direct, than it is through the cavity *b* where it is inherited from strong coupling. The parameter used are *g* = 14 meV, *g*_JC_ = 0.14 meV, *γ*_*a*_ = *γ*_*b*_ = 0.23 meV, *P*_*σ*_ = 0.1 meV, *γ*_*σ*_ = 0.001 meV, *P*_*θ*_ = 0.08 meV, *γ*_*θ*_ = 100 meV and *T* = 100 K. (c) Sketch of the correlated character of the dissipative coupling that makes the cavity emits in an environment whose configuration permits or, on the contrary, impedes its strong coupling.

There is an excellent qualitative agreement between our simple minimalistic model and the experimental data, with all the notable features being reproduced. Figure 8, for instance, gives an overview of the spectral features as a function of increasing phonon-induced coupling *γ*_*θ*_, showing the neat transition from a conventional Rabi doublet for the PM in presence of a sharp and dominating QD line at low pumping, to a triplet with the added W line and a weakening contribution from the QD line at high pumping, as is observed experimentally (cf. Figure 3). Similarly, Figure 9 shows how the temperature dependence matches with the experimental observation in Figure 4. The thermal dependence has been incorporated through the refractive index in QD-embedded PhC cavities, as described by Blakemore [72], and the Varshni model [52], [73]. Specifically, the frequency *ω*_*σ*_(*T*) = *E*_*g*_(0) − *αT* ^2^/(*T* + *β*), where *E*_*g*_ represents the gap energy of the material in which the QD is grown, and *α* and *β* are material-dependent constants. Additionally, the cavity frequency is taken as *ω*_*a*_(*T*) = *ω*_*a*_(0)/(1 + *a*′*T*), where *a*′ is a constant dependent on the materials constituting the cavity. This is similar for *ω*_*b*_(*T*). In principle, the phonon-assisted rates *γ*_*θ*_ and *P*_*θ*_ depend on temperature, detuning and the spectral density as can be demonstrated by considering the microscopic description within the Lindblad master equation approach. In fact, the phonon-assisted rates evidence a thermal dependence like an S-shape, as was shown in works explaining the mode-pulling phenomenon in cQED [71], [74]. Taking these elements into account, we have treated the parameter *γ*_*θ*_ as an S-shape in the theoretical model. In Figure 10, the linewidths dependence are contrasted, with a good qualitative agreement for pulsed excitation. While there can be some quantitative differences, these are probably due to the fact that in the actual experiment, the key variables *P*_*σ*_ and *γ*_*θ*_ are expected to be interconnected, while in the model, they are independent free parameters that we typically vary one at a time. In these conditions, it would be time consuming to aim for a fit of the data, that is also not guaranteed to be excellent since we have privileged a simple phenomenological model to capture the physics involved. In contrast, a more accurate but possibly also more confusing full-semiconductor model, should provide such a quantitative agreement. In our current modeling, we emphasize that the pump *P*_*σ*_ does not directly change the phonon-mediated coupling rates *P*_*θ*_ and *γ*_*θ*_, but rather modifies the effective environment in which the QD and cavity interact, justifying these parameters to vary as well. In any case, the theoretical model describes the characteristic and distinctive features observed in the experiment. This suggests that the anomalous W peak is due to a coexistence of weak and strong coupling, without either case overtaking the other. This gives rise to a new regime of light–matter interactions with strong qualitative hallmarks, that have been observed thanks to the versatility and richer environment provided by a solid-state platform. We have furthermore focused here on the observables brought forward by the experiment, but this simple platform of one two-level system coupled to two cavities gives rise to considerable variations in both couplings and drivings as well as in what to measure. For instance, the cavity emission was taken as the incoherent sum of both cavities, which is the most likely scenario given our detection geometry. Theoretically, however, we observe that the anomalous peak W is visible in one cavity only, namely cavity *b* (the one not coupling directly to the QD). This can be understood by looking at the emission spectrum in logaritmic scale, as shown in Figure 11. The correlated transfer of photons from the cavity *a* to the two-level system further manifests itself by a joint depletion of photon emission as well as a significant increase of the QD emission, as compared to cavity *b.* There, the QD emission is only seen indirectly from the strong coupling, and the added overall losses result in weak-coupling emission, the QD not being directly or strongly part of this cavity’s dynamics. This phenomenology and the coexistence of various mechanisms as probed differently by different modes of the system, is in agreement with our picture and provides further evidence of its realization, as well as predictions for future or further experimental investigations of this regime. A detailed analysis of the comprehensive set of other possible configurations is beyond the scope of the present work but certaintly invites a thorough analysis of the QED of a two-level system coupled to two cavities, and to pay closer attention to dissipative coupling in any system.

## 5 Conclusions

We have studied the rich phenomenology that occurs in solid-state cQED experiments involving a QD coupled to a photonic molecule. We conducted a comprehensive experimental characterization of the structure using several techniques and in various regimes of excitation. At high pumping, we observed an unexpected peak W that is energetically between the PM Rabi doublet. This peak, that bears all the features of a cavity mode, is explained by a simple phenomenological model of light–matter coupling between the QD and the PM that involves a type of correlated dissipative coupling, which is likely phonon-mediated in origin. This makes the molecule emits in two distinctive environments, that allow or on the opposite impede its strong coupling. This results in a coexistence of both regimes, as is described theoretically by a simple model that reduces the problem to its key ingredients. From this model, we can identify which elements are necessary from those that do not alter the phenomenological observation. For instance, the phonon-assisted Liouvillian coupling terms $L_{\sigma^{†}a}$ and $L_{\sigma a^{†}}$ , describing incoherent, but correlated, transfers of excitation, are required, as mere rate equations do not reproduce this dynamical dependency of the molecule’s emission on the state of the QD. Without correlations, only one regime is observed at a time, in which case a triplet would be observed if each regime could be established for long periods of time as compared to the system’s dynamics, but short as compared to the integration time, as previously discussed in the literature [57]. Our observations show how the richer and highly tunable geometries that are made possible by solid-state microcavity QED can give rise to new regimes of light–matter interactions that bring curious variations on otherwise familiar themes.
